# Protocol to analyze changes in hippocampal neural stem cell quiescence from single-cell RNA sequencing data

**DOI:** 10.1016/j.xpro.2025.104226

**Published:** 2025-11-24

**Authors:** Jessica Hart, Lachlan Harris

**Affiliations:** 1 QIMR Berghofer, Brisbane, QLD 4006, Australia; 2 The University of Queensland, Brisbane, QLD 4067, Australia; 3 Queensland University of Technology, Brisbane, QLD 4059, Australia

**Keywords:** Developmental biology, Cancer, Neuroscience, Stem cells

## Abstract

Here, we present a protocol to isolate mouse hippocampal neural stem cells from single-cell RNA sequencing datasets using a computational approach. We describe steps to analyze whether a perturbation affects quiescence depth. Specifically, we detail procedures for sequential reclustering, pseudotime trajectory analysis, and statistical testing of distribution shifts from deep to shallow quiescence. This protocol overcomes challenges in distinguishing quiescent neural stem cells from astrocytes and proliferating neural stem cells from progenitor cells.

For complete details on the use and execution of this protocol, please refer to Rigo et al.^1^

## Highlights

• Instructions for isolating quiescent stem cells from mouse hippocampal scRNA-seq
• Steps for separating quiescent neural stem cells from astrocytes
• Guidance on separating proliferating neural stem cells from progenitors
• Procedures for ordering quiescence of neural stem cells via trajectory inference

## Abstract (editor highlights)

Publisher’s note: Undertaking any experimental protocol requires adherence to local institutional guidelines for laboratory safety and ethics.

## Graphical abstract

**Figure:**
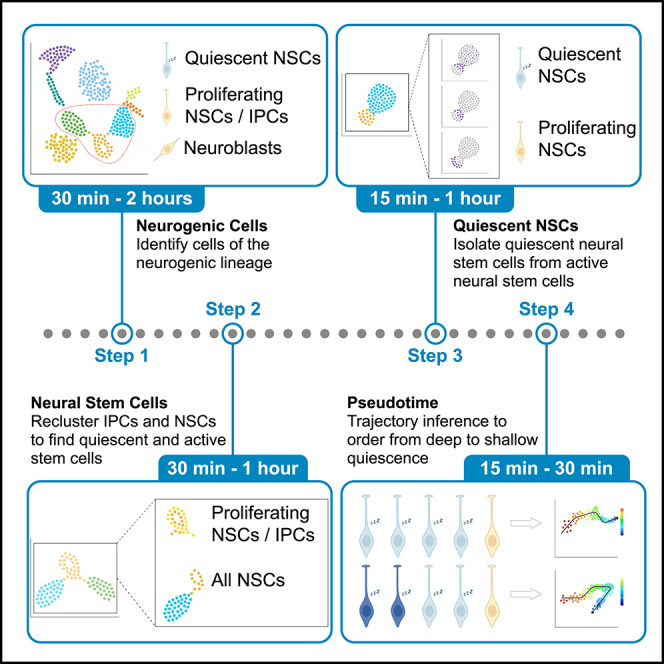

## Before you begin

This protocol outlines biologically-informed computational steps for determining the effect of genetic and physiological perturbations on the quiescent states of adult mouse hippocampal neural stem cells (NSCs) via single-cell RNA-sequencing (scRNA-seq). The example dataset features a genetic mouse mutant, in which the *Mycn* gene was deleted from *Glast* expressing cells, including adult hippocampal NSCs at 1-month of age, causing cells to stall between deep and shallow states of quiescence. The cells sequenced in this study were derived from dissection and enzymatic dissociation of the dentate gyrus and isolation of Nestin-GFP^+^ cells by flow sorting. These cells were then processed by 10× Genomics 3′ scRNA-seq.^1^ However, the protocol can be adapted for use with other methods of enriching hippocampal neural stem and progenitor cells (e.g., Glast-creERT2; Rosa-YFP) and with other genetic or experimental conditions affecting quiescence, such as aging.^2^^,^^3^ Likewise, similar analytical principles can be applied to scRNA-seq data from the subventricular zone (SVZ), although marker genes may differ, for example *Hopx* is expressed only by hippocampal NSCs.^4^ The approach can also be applied to single-nucleus RNA-sequencing data without significant modifications.

### Innovation

Two key challenges addressed here include how to distinguish quiescent NSCs from mature astrocytes, due to substantial transcriptomic overlap between these cells. Similarly, proliferating NSCs often cluster with intermediate progenitor cells (IPCs) due to the fact that these cells are both progressing through the cell-cycle, which causes extensive and concordant transcriptomic changes. These challenges complicate accurate characterization of NSC dynamics during adult neurogenesis. The key innovation is that our protocol introduces a multi-step filtering approach combining gene expression markers with the observation that proliferating IPCs and NSCs form distinct rings in UMAP space. This enables more accurate separation of quiescent and proliferating NSCs for downstream analyses.

### Hardware preparation

1. Perform the analysis in R, ideally using RStudio, on Linux, macOS, or Windows operating systems.

***Note:*** We recommend a system with at least 32 GB of RAM and 4 CPU cores. On a high-performance computing cluster, request equivalent resources.

### Software preparation

2. Install the key software packages listed in the key resources table. For example:

> install.packages("Seurat")

***Note:*** Although using different software versions (e.g., Seurat) and computational environments will alter data visualization, the fundamental analytical approaches remain stable. The goal of this protocols paper is to demonstrate the key principles we have employed to isolate hippocampal NSCs from scRNA-seq data. Therefore, when implementing this protocol, especially for different datasets, follow the biologically-informed guidance and alter code accordingly (e.g., the clusters to isolate).

### Setting up the computational environment

**Timing:10–15 min**

3. Launch RStudio and create a new project named ‘Mycn_NSC_analysis’. Install the necessary packages listed in the key resources table.
4. Download count matrices from GEO (accession GSE280296). Obtain the following samples: *Mycn* control replicate 1 (GSM8594261), *Mycn* control replicate 2 (GSM8594263), *Mycn* conditional knockout (cKO) replicate 1 (GSM8594260), and *Mycn* cKO replicate 2 (GSM8594262). Create a folder named "data” within your RStudio project directory and move the downloaded count matrices into this folder.

## Key resources table

**Table:** 

REAGENT or RESOURCE	SOURCE	IDENTIFIER
**Deposited data**		

GSE280296	Rigo et al.^1^	https://www.ncbi.nlm.nih.gov/geo/query/acc.cgi?acc=GSE280296

**Software and algorithms**		

R v.4.5.0	R Development Core Team	https://www.r-project.org/
RStudio	RStudio	https://posit.co/download/rstudio-desktop/
Seurat (v.5.3.0)	Hao et al.,^5^ CRAN	https://cran.r-project.org/web/packages/Seurat/
tidyverse (v.2.0.0)	CRAN	https://cran.r-project.org/web/packages/tidyverse/
RColorBrewer (v.1.1.0)	CRAN	https://CRAN.R-project.org/package=RColorBrewer
Slingshot (v.2.16.0)	Street et al.^6^ part of Bioconductor	https://www.bioconductor.org/packages/release/bioc/html/slingshot.html
SingleCellExperiment (v.1.30.1)	Amezquita et al.^7^ part of Bioconductor	https://bioconductor.org/packages/release/bioc/html/SingleCellExperiment.html
R.utils (v.2.13.0)	CRAN	https://cran.r-project.org/web/packages/R.utils/
Custom code	Zenodo (this study and Rigo et al.^1^)	https://doi.org/10.5281/zenodo.17255383

**Other**		

Computational resources	High-performance computer with 32 GB of RAM and 4 CPUs	

## Step-by-step method details

***Note:*** All scripts used in this analysis are available on Zenodo (see **Data and code availability**). At the end of each script, the Seurat object is saved. It is then read in at the start of the next script. This was designed so that researchers can pause at each step, if needed.

### Isolation of neurogenic clusters

**Timing:30 min–2 h**

1. Read the four scRNA-seq samples and merge them into a single Seurat object.
  a. Go to the tutorials folder and open the “01_read_merge.R” script.
  b. Run the code in the R script.

***Note:*** In this script, the workflow reads and merges scRNA-seq data from four *Mycn* datasets (2 control and 2 knockout replicates) from GEO, merges them into a single Seurat object, performs quality control filtering and adds metadata, including experimental batch and mouse genotype.

2. Normalize and integrate the four experimental samples according to experimental replicate (batch).
  a. Go to the tutorials folder and open the “02_normalise_integrate_neurogenic.R” script.
  b. Run the code in the R script.

***Note:*** SCTransform is one of two main data normalization workflows in Seurat.^5^ This script performs batch-corrected integration on all cells from the preprocessed *Mycn* dataset and visualizes marker genes to isolate cell clusters belonging to the neurogenic lineage.

**CRITICAL:** Batch effects are typically strong in scRNA-seq data. Providing the replicates are balanced, which is to say in each replicate a control and experimental condition is present, replicates can be “integrated” according to their batch of origin. With this experimental design, this can remove technical effects that are batch-dependent, while preserving the biological effect.^8^ If integration does not sufficiently overcome batch effects, consider if it is appropriate to independently perform quality control in a batch-dependent manner. Between Seurat v4 and v5 the integration workflow changes: in v5 PCA (on unintegrated data) is run before integration, though the downstream results are broadly similar, be sure to use the workflow that suits your Seurat version.

3. Isolate three neurogenic clusters from the resulting Seurat object (i.e., quiescent NSCs, proliferating NSCs/IPCs and immature neurons).
  a. Visualize gene expression of key genes to isolate a quiescent NSC cluster, a combined proliferating NSC and IPC cluster and non-cycling immature neuron cluster.

**Figure 1:**
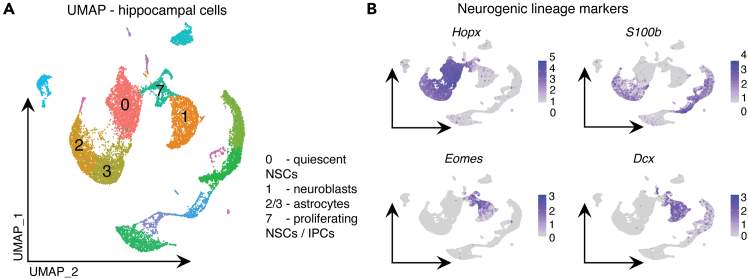
Isolation of neurogenic clusters derived from mouse hippocampus (A) UMAP of Nestin-GFP^+^ cells isolated from the mouse dentate gyrus of *Mycn* control and *Mycn* cKO mice and processed for scRNA-seq. (B) Gene expression patterns for *Hopx*, *S100b*, *Eomes* and *Dcx* enable the identification of neurogenic cells in clusters 0 (quiescent NSCs), 1 (immature neurons/neuroblasts) and 7 (proliferating NSCs and IPCs).

***Note:*** The quiescent NSC cluster (*Hopx*-high; *S100b*-low)^9^ is distinguished from astrocytes that are *Hopx*-low; *S100b*-high.^10^^,^^11^ See limitations section for discussion. The combined proliferating NSCs and IPC cluster has a circular topology and appears predominantly as *Eomes*-high, though is a mixed population.^12^ Due to major transcriptional changes that occur upon cell-cycle entry, proliferating NSCs end up clustering with IPCs and away from quiescent NSCs. These proliferating NSCs are recovered in the next stage of the protocol. The non-cycling immature neuron cluster is *Dcx*-high; *Eomes*-low. In the example below, cells to isolate belong to clusters “0”, “1”, and “7” (Figure 1). Other types of cells found in the dataset include microglia and immature and mature oligodendrocytes.

# Define the key genes for visualisation

ngenic_ident <- c("Hopx", "S100b", "Eomes", "Dcx")

# Generate feature plots for each of the key genes

final.integrated <- NormalizeData(final.integrated, assay = "RNA")

DefaultAssay(final.integrated) <- "RNA"

feature_plots <- FeaturePlot(object = final.integrated, features = ngenic_ident, reduction = "umap", dims = c(2, 1), pt.size = 1)

Fig1B <- feature_plots

ggsave("plots/Figure_1B.png", plot = Fig1B, dpi = 300, height = 7, width = 10)

# Generate the dimensionality reduction plot with cluster labels

dim_plot <- DimPlot(final.integrated, label = TRUE, reduction = "umap", dims = c(2, 1), pt.size = 1)

# Compare plots side-by-side

dim_plot + feature_plots

# Subset the populations to a new Seurat Object named final.integrated

final.integrated <- subset(final.integrated, idents = c("0", "1", "7"))

### Isolation of neural stem cells

**Timing:30 min–1 h**

4. Renormalize and integrate the four experimental samples, now containing only cells in the neurogenic lineage.
  a. Go to the tutorials folder and open the “03_normalise_integrate_neuralstemcells.R” script.
  b. Run the code in the R script.

***Note:*** This script performs batch-corrected integration on all neurogenic cells, removes contaminating clusters and isolates NSCs by selecting cells with expression of *Hopx* or *Apoe*.

5. Isolate NSCs from the resulting Seurat object. Quiescent NSCs already cluster separately, but proliferating NSCs are intermingled with IPCs.
  a. Visualize gene expression of key markers such as *Hopx, Apoe, Eomes, Dcx* and *S100b* to identify proliferating NSCs and distinguish them from IPCs.

**Figure 2:**
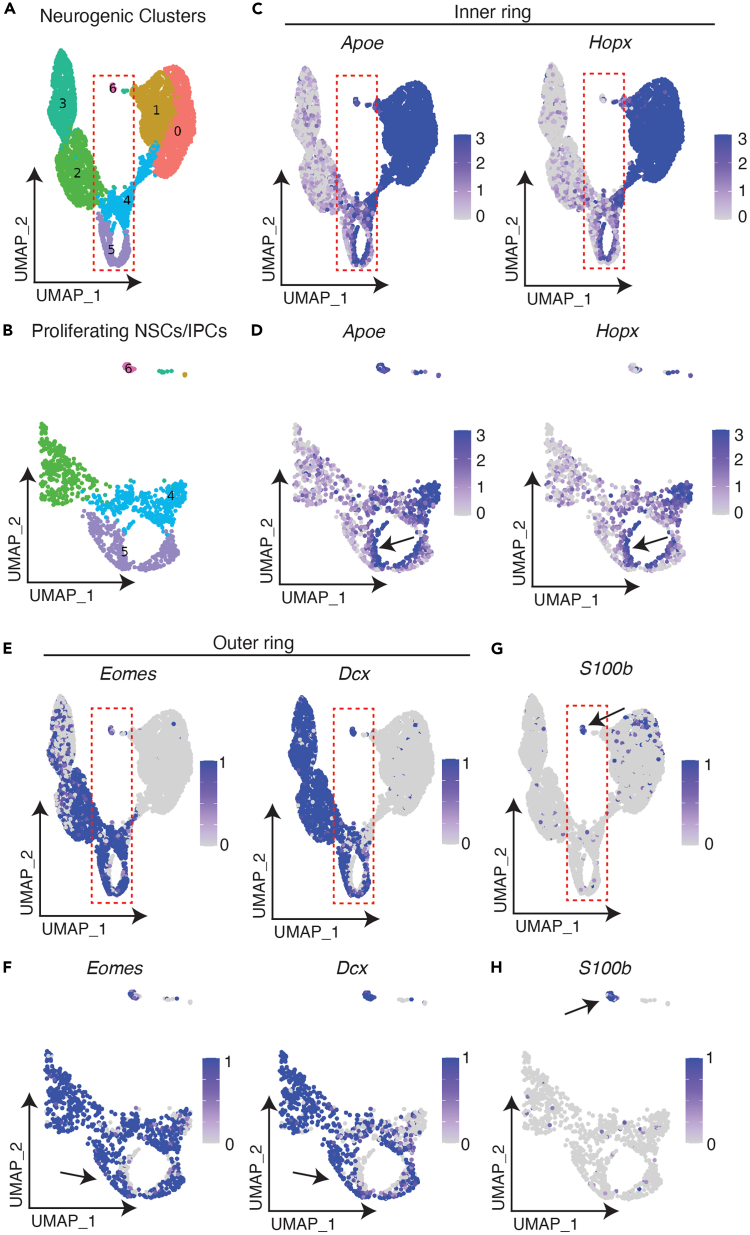
Isolation of quiescent and proliferating NSCs (A) UMAP of neurogenic populations after reclustering. Quiescent NSCs belong to clusters 0 and 1; proliferating NSCs are clustered with IPCs within clusters 4 and 5. (B) UMAP of boxed region in (A). (C) Proliferating NSCs form an inner ring of *Apoe*/*Hopx-*expressing cells. (D) Zoomed-in region from panel C. Arrow indicates inner ring of proliferating NSCs. (E) IPCs form an outer ring of *Eomes* and *Dcx-*expressing cells. (F) Zoomed in region from panel E. Arrow indicates outer ring of IPCs. (G) UMAP showing *S100b* expression, with arrow indicating likely multiplet cluster expressing *S100b* and *Dcx*. (H) Zoomed-in region from panel G.

***Note:*** Proliferating NSCs form an inner ring characterized by *Hopx* and absent *Eomes* expression (Figure 2); IPCs show low *Hopx* and high *Eomes* expression. In the example below, to isolate all NSCs, we isolate cells with high *Hopx* or high *Apoe* expression. Thresholds for gene expression selection are explicitly defined in the script “03_normalise_integrate_neuralstemcells.R” but should be adjusted according to the dataset being used, as appropriate. The thresholds are not strictly quantitative; rather, they are tuned to achieve the cleanest isolation of the inner ring of proliferating NSCs and exclusion of the outer ring of *Eomes*-expressing IPCs.

# Generate full DimPlot and FeaturePlots

DefaultAssay(final.integrated) <- "RNA"

dim_plot_full <- DimPlot(final.integrated, label = TRUE, reduction = "umap", pt.size = 1) + NoLegend()

apoe_plot_full <- FeaturePlot(final.integrated, features = "Apoe", reduction = "umap", max.cutoff = 3, pt.size = 1)

hopx_plot_full <- FeaturePlot(final.integrated, features = "Hopx", reduction = "umap", max.cutoff = 3, pt.size = 1)

eomes_plot_full <- FeaturePlot(final.integrated, features = "Eomes", reduction = "umap", max.cutoff = 1, pt.size = 1)

dcx_plot_full <- FeaturePlot(final.integrated, features = "Dcx", reduction = "umap", max.cutoff = 1, pt.size = 1)

s100b_plot_full <- FeaturePlot(final.integrated, features = "S100b", reduction = "umap", max.cutoff = 1, pt.size = 1)

# Generate cropped DimPlot and FeaturePlots

# Note you may need to adjust the co-ordinates depending on your plot

dim_plot_crop <- dim_plot_full + xlim(-6, 0.8) + ylim(-9, 4)

apoe_plot_crop <- apoe_plot_full + xlim(-6, 0.8) + ylim(-9, 4)

hopx_plot_crop <- hopx_plot_full + xlim(-6, 0.8) + ylim(-9, 4)

eomes_plot_crop <- eomes_plot_full + xlim(-6, 0.8) + ylim(-9, 4)

dcx_plot_crop <- dcx_plot_full + xlim(-6, 0.8) + ylim(-9, 4)

s100b_plot_crop <- s100b_plot_full + xlim(-6, 0.8) + ylim(-9, 4)

# Arrange full and cropped plots side-by-side for comparison of gene expression levels

Fig2 <- (dim_plot_full | apoe_plot_full | hopx_plot_full | eomes_plot_full | dcx_plot_full | s100b_plot_full) /

 (dim_plot_crop | apoe_plot_crop | hopx_plot_crop | eomes_plot_crop | dcx_plot_crop | s100b_plot_crop)

Fig2

ggsave("plots/Figure_2.png", plot = Fig2, dpi = 300, height = 8, width = 20)

### Isolation of quiescent neural stem cells

**Timing:15 min–1 h**

6. Renormalize and integrate the four experimental samples, now containing only quiescent and proliferating NSCs.
  a. Go to the tutorials folder and open the “04_normalise_integrate_quiescentneuralstemcells.R” script.
  b. Run the code in the R script.

***Note:*** This script performs a third round of batch-corrected integration on the NSC population and removes proliferating NSCs, to isolate only the quiescent NSCs.

**Figure 3:**
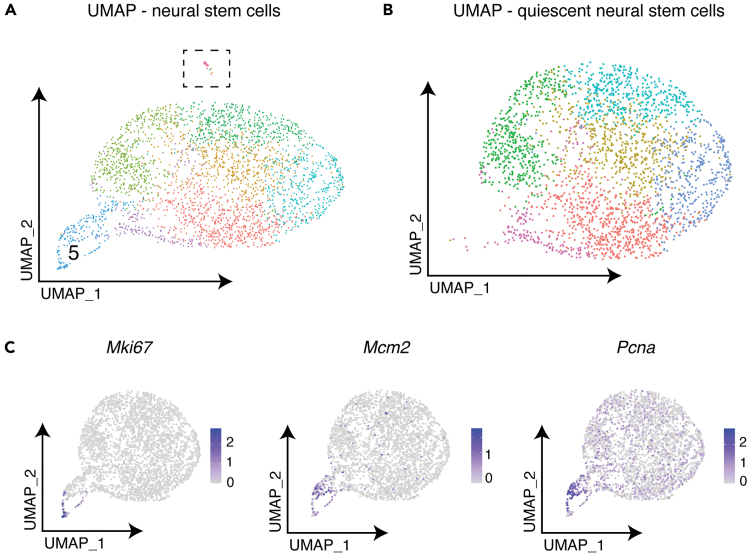
Isolation of quiescent NSCs (A) UMAP of NSCs showing predominantly quiescent cells with a small proliferating population in cluster 5, which exhibits a circular topology. Dashed box highlights contaminating cells expressing neuroblast markers that require removal. (B) UMAP of quiescent NSCs after removal of proliferating NSCs and contaminating neuroblasts. (C) Expression of proliferation markers (*Mki67*, *Mcm2* and *Pcna*), confirming the proliferating state of cluster 5 cells.

7. Isolate quiescent NSCs by excluding the cluster with circular topology expressing high levels of *Mki67* and other proliferation markers (Figure 3).

# Remove final erroneous cluster of cells

# comprising cluster of scattered cells that do not cluster with remainder of NSCs and have high neuroblast gene expression

p1 <- DimPlot(final.integrated)

cells_to_remove <- CellSelector(p1)

final.integrated <- subset(final.integrated, cells = setdiff(Cells(final.integrated), cells_to_remove))

############################################

# To visualise which clusters to subset

############################################

p2 <- FeaturePlot(final.integrated, features = c("Mki67", "Mcm2", "Pcna"), ncol = 3) # There are other proliferation markers

# Remove proliferating NSCs

final.integrated <- subset(final.integrated, idents = "5", invert = TRUE)

p3 <- DimPlot(final.integrated, reduction = "umap", group.by = "seurat_clusters", label = FALSE, pt.size = 1) + ggtitle("UMAP of Clusters")

# Combine side by side

p1 + p3

p2

### Pseudotime ordering of quiescent neural stem cells

**Timing:15 min – 30 min**

8. Renormalize and integrate the four experimental samples, now containing only quiescent NSCs. Next convert the final integrated SeuratObject to a SingleCellExperiment and run Slingshot.^6^
  a. Go to the tutorials folder and open the script “05_pseudotime.R”
  b. Run the code in the R script.

***Note:*** In this script, a final round of batch-corrected integration is performed on quiescent NSCs. A pseudotime trajectory is constructed via Slingshot and the distribution of cells along this pseudotime is compared between *Mycn* cKO and control genotypes using a Kolmogorov-Smirnov test.

**CRITICAL:** Do not specify clusters, run Slingshot as a single cluster as this reflects the biology of the activation process, which occurs as a single continuous process. However, in approximately half of cases, this approach results in a reversed ordering, where pseudotime begins from shallow states of quiescence and progresses towards deep quiescence. The orientation can be determined through expression of genes such as *Ccnd1*, which increase as cells move towards shallow quiescence. Code to interrogate the orientation is provided in the script. While an inversion does not meaningfully affect downstream analyses, it can be corrected for example, by specifying a starting cluster (see Slingshot documentation) to ensure the trajectory reflects the correct biological direction.

**Figure 4:**
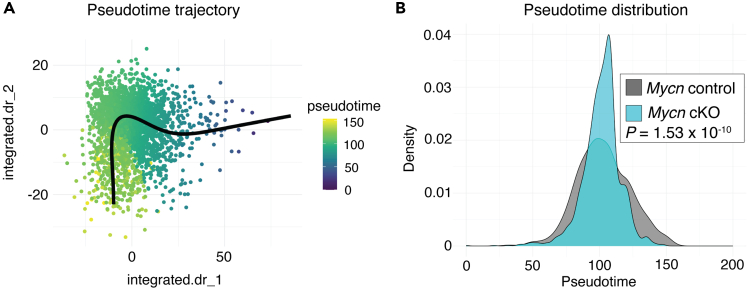
Pseudotime ordering of quiescent NSCs in *Mycn* control *and Mycn* cKO mice (A) Pseudotime trajectory of quiescent NSCs from *Mycn* control and *Mycn* cKO mice through the first two principal components of the integrated dimensional reduction. Black line indicates the principal curve generated by Slingshot. (B) Pseudotime ordering from deep to shallow quiescence shows the density estimates of pseudotime distributions of quiescent NSCs from *Mycn* control and *Mycn* cKO mice. Statistics: Kolmogorov-Smirnov test comparing distribution of quiescent NSCs across pseudotime in *Mycn* control and *Mycn* cKO mice.

***Note:*** A ks.test is used to determine if the distributions between the conditions are different, even if their means are unchanged. This test is particularly useful and the most appropriate choice when distribution shape rather than mean is altered, as exemplified by the phenotype of the *Mycn* cKO mice, whereupon cells accumulate at approximately the median pseudotime position observed in control mice. Detailed instructions for statistical testing (ks.test) are explicitly outlined in the “05_pseudotime.R” script.

9. Extract pseudotime values of control and cKO mice and perform statistical testing (Figure 4).

## Expected outcomes

We have provided visualizations demonstrating the sequential isolation of pure populations of quiescent NSCs from the scRNA-seq dataset (Figure 1). After isolating the quiescent NSCs and performing Slingshot analysis,^6^ researchers can expect to accurately order hippocampal NSCs along a pseudotime trajectory from deep to shallow quiescence. This approach enables users to assess how specific experimental interventions (such as gene deletions or aging) affect the depth of NSC quiescence.

Quantitatively, researchers will obtain pseudotime positions for each individual quiescent hippocampal NSC. It is important to consider potential variability, particularly batch effects between replicates, which require careful integration of samples. Additionally, since clusters are not explicitly specified during Slingshot analysis, the pseudotime ordering may initially invert (i.e., running from shallow to deep rather than deep to shallow). This inversion can be corrected, for example by specifying a start or end cluster.

The validity of these computational findings can be confirmed through complementary experimental approaches. For example, if a mouse mutant causes hippocampal NSCs to remain in deeper quiescent states, immunofluorescence staining of protein markers characteristic of these deeper states, such as ID4,^2^ should exhibit higher expression levels compared to controls. Furthermore, biological validation may involve assessing the production of IPCs and neuroblasts, which typically decrease when NSCs remain in deeper quiescence but increase if NSCs shift towards shallower quiescent states.

## Quantification and statistical analysis

A ks.test is used to determine if the pseudotime distributions between conditions are different, even if their means are unchanged. This is the most appropriate choice for the phenotype of the *Mycn* cKO mice, in which cells accumulate at approximately the median position of pseudotime observed in control mice. However, depending on the specific data distribution, it is recommended that researchers evaluate the assumptions of each test and choose the most suitable statistical test accordingly.

## Limitations

Several factors may limit the reliability or applicability of this protocol. Computational constraints can pose challenges, particularly when handling large datasets; therefore, analysis may require high-performance computing resources rather than local machines. However, after sequential clustering is performed and only cells of the neurogenic lineage remain, datasets typically become smaller and manageable on standard computing setups.

Biologically, accurately distinguishing all quiescent NSCs from mature astrocytes is challenging due to significant transcriptomic overlap. While genes associated with these different cell populations exist, they are typically expressed at different relative levels than in a binary on/off manner. For example, in addition to *Hopx* and *S100b*, we find that genes such as *Sparc* are enriched in quiescent NSCs, whereas *Aqp4* and *Rorb,*^13^ have increased expression in astrocytes. While separate clusters of astrocytes and NSCs appear in the data, intermingling of these populations is possible and further efforts to clearly establish the gradient of differences between these cell types is a worthwhile endeavor. Moreover, effectively distinguishing proliferating NSCs from IPCs significantly depends on obtaining a sufficient cell number, as the clarity and accuracy of these distinctions greatly improve with larger datasets.

This protocol specifically addresses pseudotime ordering of NSCs from deep to shallow quiescence states, explicitly excluding proliferating NSCs. This exclusion is necessary as the dominance of cell-cycle gene expression can overshadow subtle quiescence-related phenotypes when ordering along the main axes of gene expression variation.

Variations in bioinformatics package versions, can affect clustering outcomes and subsequent analyses. Although replicating the exact computational environment may mitigate these variations, a more practical approach involves using robust, generalizable criteria. For example, consistent patterns such as *Hopx*-high and *S100b*-low expression in NSCs versus *Hopx*-low and *S100b*-high in astrocytes remain reliable indicators, even if cluster numbers vary.

## Troubleshooting

### Problem 1 (related to numerous steps for cluster visualization)

Insufficient cluster resolution used to identify cell populations.

### Potential solution

Cluster resolutions that are too low can lead to clusters that are too broad and therefore it can be difficult to distinguish cell types. When choosing cluster resolution, we recommend that you consider the gene expression profiles described in the protocol, these genes allow for the discernment of the populations: NSCs, IPCs and neuroblasts. The cluster resolutions used in the various scripts are reasonable starting points, packages such as clustree can also be used to visualize stable clusters.^14^

### Problem 2 (related to step 5)

Inability to distinguish proliferating NSCs from IPCs.

### Potential solution

In our experience, the clear identification of this inner-ring of NSCs, which cluster with IPCs, is dependent on having sufficient numbers of cells sequenced. As a starting point we sequenced 6,784 – 12,548 cells in each of the mouse mutant strains described in our study, with 9,187 cells sequenced in the example for this protocols paper.^1^ If clear partitioning of these cell types is not possible in the analyzed dataset, consider acquiring more cells for sequencing.

### Problem 3 (related to step 7)

Impaired visualization of cell cycle activity due to cell-cycle regression.

### Potential solution

In some standard scRNA-seq analysis pipelines, it is suggested to perform cell cycle regression to remove cell-cycle genes that contribute to variation. Although we have not tested these effects, as we are explicitly interested in cell-cycle effects, we advise avoiding this as it might obscure distinctions between quiescent and proliferating NSCs.

### Problem 4 (related to numerous steps including data subsets)

Clear batch or replicate effects in sequencing data.

### Potential solution

Providing the data analyzed are balanced, so that treatment and control groups are present in each replicate, the data can be integrated to control for these effects. The data will need to be integrated at each iteration of subset and reclustered.

### Problem 5 (related to before you begin: Step 1)

Insufficient computing resources requested.

### Potential solution

It can be difficult to estimate computing resources required, if this occurs in early processing steps that are more computationally intensive, request more computing resources.

## Resource availability

### Lead contact

Further information and requests for resources and reagents should be directed to and will be fulfilled by the lead contact, Lachlan Harris (lachlan.harris@qimrb.edu.au).

### Technical contact

Technical questions regarding the execution of this protocol should be directed to and will be answered by the technical contact, Lachlan Harris (lachlan.harris@qimrb.edu.au).

### Materials availability

This study did not generate new unique reagents or materials.

### Data and code availability

The datasets analyzed during this protocol are publicly available on the Gene Expression Omnibus (GEO) under accession number GSE280296. All code used to generate and analyze these datasets is publicly available on Zenodo at https://doi.org/10.5281/zenodo.17255383.

## Acknowledgments

J.H. is supported by an Australian Government Research Training Program (RTP) Scholarship, funded by the Department of Education. L.H. was supported by an NHMRC Investigator grant (GNT2017476). This work was also supported by QIMR Berghofer and the Australian Cancer Research Foundation through infrastructure granted to the ACRF Centre for Optimised Cancer Therapy. The graphical abstract was created in BioRender (Harris, L. [2025] https://BioRender.com/ciyfl7p). We thank Sara Ahmed-de-Prado, Piero Rigo, Rebecca L. Johnston, and François Guillemot for useful discussions that led to this protocols paper.

## Author contributions

L.H. conceptualized the protocol, performed validation, wrote the original draft, reviewed and edited the manuscript, supervised the project, and acquired funding. J.H. contributed to validation, writing of the original draft, and review and editing of the manuscript.

## Declaration of interests

The authors declare no competing interests.

## Declaration of generative AI and AI-assisted technologies in the writing process

During the preparation of this work, the authors used ChatGPT (OpenAI, versions 4 and later) in order to assist in editing text originally drafted by the authors, with the aim of improving clarity and readability. After using this tool/service, the authors reviewed and edited the content as needed and take full responsibility for the content of the publication.
